# Oxidative Stress and Cellular Response to Doxorubicin: A Common Factor in the Complex Milieu of Anthracycline Cardiotoxicity

**DOI:** 10.1155/2017/1521020

**Published:** 2017-10-18

**Authors:** Donato Cappetta, Antonella De Angelis, Luigi Sapio, Lucia Prezioso, Michela Illiano, Federico Quaini, Francesco Rossi, Liberato Berrino, Silvio Naviglio, Konrad Urbanek

**Affiliations:** ^1^Department of Experimental Medicine, Section of Pharmacology, University of Campania Luigi Vanvitelli, Naples, Italy; ^2^Department of Biochemistry, Biophysics and General Pathology, University of Campania Luigi Vanvitelli, Naples, Italy; ^3^Department of Medicine and Surgery, University of Parma, Parma, Italy

## Abstract

The production of reactive species is a core of the redox cycling profile of anthracyclines. However, these molecular characteristics can be viewed as a double-edged sword acting not only on neoplastic cells but also on multiple cellular targets throughout the body. This phenomenon translates into anthracycline cardiotoxicity that is a serious problem in the growing population of paediatric and adult cancer survivors. Therefore, better understanding of cellular processes that operate within but also go beyond cardiomyocytes is a necessary step to develop more effective tools for the prevention and treatment of progressive and often severe cardiomyopathy experienced by otherwise successfully treated oncologic patients. In this review, we focus on oxidative stress-triggered cellular events such as DNA damage, senescence, and cell death implicated in anthracycline cardiovascular toxicity. The involvement of progenitor cells of cardiac and extracardiac origin as well as different cardiac cell types is discussed, pointing to molecular signals that impact on cell longevity and functional competence.

## 1. Introduction

Cardiovascular diseases and cancer represent the first and second cause of death in industrialized countries. These two conditions may become synergistic if we consider the cardiovascular complications of anticancer therapies.

During the past three decades, the development of effective screening and treatment strategies for many neoplastic diseases has resulted in an enormous population of long-term cancer survivors: it is currently estimated that five-year-survival rates involve over the 84% of children and 67% of the whole population diagnosed with cancer [1]. As for other chronic conditions, cancer is considered a curable disease; thus, the concerns about long-range quality of life of oncologic patients are an important emerging issue. Therefore, in the last years, the awareness and recognition of the importance of cardiotoxic side effects of anticancer therapies have increased. Recently, expert panels in both Europe and the United States have issued practice guidelines on surveillance and management strategies aimed at caring for cancer patients at high risk of cardiovascular events [2, 3].

## 2. Doxorubicin Cardiotoxicity

Discovered in Italy over a half century ago, anthracyclines continue to represent a pillar of many cancer treatment protocols. These agents, among several classes of chemotherapeutics, are drugs that more frequently induce cardiotoxic effects. Although anthracycline-induced cardiotoxicity constantly stimulates substantial interest of basic and clinical researchers, despite efforts, the precise mechanism of this complication remains unclear.

Cardiac dysfunction triggered by doxorubicin (DOX) has long been known as the main form of anticancer drug-induced cardiotoxicity, being characterized by massive accumulation of reactive oxygen species (ROS) and reactive nitrogen species (RNS) as central mechanisms [4–6]. The pharmacokinetics of DOX, that accumulates within the myocardium, together with low level of antioxidant enzymes in cardiomyocytes can be viewed as permissive conditions for cardiotoxicity. High level of ROS and RNS may activate cytotoxic signalling leading to DNA damage, mitochondrial dysfunction, attenuation in protein synthesis, and deregulation of intracellular calcium homeostasis [7–10]. From a cellular point of view, several enzymes, including xanthine oxidases, NADPH oxidases (Noxs), uncoupled nitric oxide (NO) synthases (NOSs), and peroxisomes, located in subcellular compartments, such as mitochondria, sarcoplasmic reticulum, and cytoplasm, account as a source of ROS [11]. Generally, mitochondria are the site where most of the ROS are produced. ROS-producing enzymes within mitochondria can transform DOX to semiquinone through one-electron reduction of the quinone moiety in ring C. This semiquinone can readily react with oxygen-generating superoxide anion (O_2_∙^−^), which could be neutralized into relatively stable and low-toxic hydrogen peroxide (H_2_O_2_) by superoxide dismutase, or further changed to other ROS or RNS in a sequence of reactions known as the redox cycling. Dangerously, H_2_O_2_ and O_2_∙^−^ may also generate highly reactive and toxic hydroxyl radicals (OH∙) during the iron-catalysed Haber-Weiss reaction [7, 12, 13]. The strict connection between anthracyclines and mitochondria lays in the high affinity for cardiolipin, a phospholipid located in the inner mitochondrial membrane, where anthracyclines, being retained at higher concentration, disrupt electron-transport chain thus inducing more ROS production [14]. Mitochondria are also the site where upregulated MnSOD increase cell survival in the presence of DOX, exerting its role as a free radical scavenger [15].

NOS is another major source of DOX-dependent ROS. DOX is able to determine the increase in the expression of endothelial NOS (eNOS) and, by a direct binding, to interfere with NO generation in favour of superoxide formation [16, 17]. ROS, reacting with NO to generate RNS, boost the generation of oxidants that, in turn, force the uncoupling of eNOS into monomers, altering the enzymes' function to produce more superoxide anion and less NO. The pivotal role of eNOS in DOX-dependent oxidative stress was confirmed in a study conducted on eNOS knockout mice that showed low levels of ROS and preserved myocardial function after exposure to DOX. On the contrary, the cardiomyocyte-specific overexpression of eNOS enhanced the detrimental effects of DOX on the heart [18]. DOX treatment seems to modulate also the activity of the other isoform, inducible NOS (iNOS), but its role in the pathogenesis of anthracycline cardiomyopathy has not been fully elucidated. Indeed, conflicting results about the cardiotoxic effect of DOX were observed in studies using iNOS^−/−^ mice, which reported both amplified and reduced level of cell damage [19, 20].

NADPH oxidase-dependent ROS accumulation is strongly influenced by DOX. Nox2 and Nox4 are the predominant isoforms expressed in the heart where under pathological stimuli they contribute to enhance oxidative stress by reducing oxygen to superoxide anion and hydrogen peroxide with the support of NADPH as an electron donor [21]. Many evidence indicated Nox2-derived ROS to have a key role in DOX-induced toxicity, both *in vitro* and *in vivo* [22–24]. Genetic disruption of Nox2 attenuated DOX-related myocardial dysfunction, with less myocardial atrophy, cardiomyocyte apoptosis, and interstitial fibrosis [25]. The association between cardiotoxicity and gene variants in the subunits of human NADPH oxidases reinforces the relevance of these enzymes in anthracycline cardiomyopathy [26, 27].

Redox cycling of DOX is not the unique phenomenon at the base of cardiotoxicity. Anticancer activity of DOX is mainly attributed to its well-known ability to bind topoisomerase 2 (Top2, in particular, the *α* isoform) and DNA in cancer cells leading to cell death. However, also other isoforms of Top can interact with DOX. Cardiomyocytes express nuclear (the *β* isoform of Top2) and mitochondrial topoisomerases that are targeted by DOX [28, 29]. Recent studies showed that cardiomyocyte-specific deletion of Top2-*β* protects the heart from DOX damage mainly by interfering with a defective mitochondrial biogenesis and ROS formation [30]. According to the Top2-*β* hypothesis, the interaction between anthracycline and Top2-*β* accounts as the initial event of cardiotoxicity that prompts ROS accumulation as a subordinate step. Whatever the case, as either originating or downstream event, oxidative stress maintains its key importance in the pathogenesis of anthracycline cardiomyopathy and its modulation is still highly attractive from a molecular and therapeutic perspective.

## 3. Beyond Cardiomyocytes: Expanded Repertoire of Cellular Targets

Numerous molecular elements have been implicated in the pathogenesis of DOX cardiotoxicity but it is clear that, up to date, a single and unified model of pathogenesis of this disease has not been formulated. Proper myocardial hemodynamic function is maintained upon an adequate oxidative energy production in billions of individual cardiomyocytes in an adult human heart. In this regard, ROS formation and involvement of mitochondria as a subcellular target of DOX are typically studied in the context of a cardiomyocyte as a cell type particularly rich in mitochondria. Commonly to an antitumoral action on cancer cells [31–35], it is generally accepted that oxidative stress leads to the activation of necrotic and apoptotic pathways causing loss of cardiomyocytes [7, 36–38]. Convincing results on the entity and incidence of apoptosis raised the possibility that apoptotic-related mechanisms are central in the setting of acute cardiotoxicity but less important in a scenario of chronic cardiomyopathy and heart failure. Increasing evidence indicate that other mechanisms, including senescence, take part in anthracycline-driven cardiotoxic effects, affecting the functional activity of cardiomyocytes and other cardiac cells. Senescence consists of growth arrest of normal somatic and postmitotic cells with a consequent reduction in function and organ damage [39]. Short exposure of cells to subcytotoxic concentrations of oxidants or DNA-damaging agents could also lead to cellular senescence [40]. Indeed, low concentrations of DOX can induce terminal growth arrest with senescence-like alterations in proliferating cells [41]. Interestingly, the changes in the expression of several aging-related genes in cultured neonatal cardiomyocytes were similar to those observed in response to DOX. Telomerase activity is detectable not only in proliferating cells but also in cardiomyocytes, playing an important role against cell death [42]. In particular, DOX-treated neonatal cardiomyocytes show the reduction in telomerase activity, telomere length, and the telomerase reverse transcriptase protein level, in a time-dependent manner as experienced by cardiomyocytes of an aged rat heart [43].

**Figure 1:**
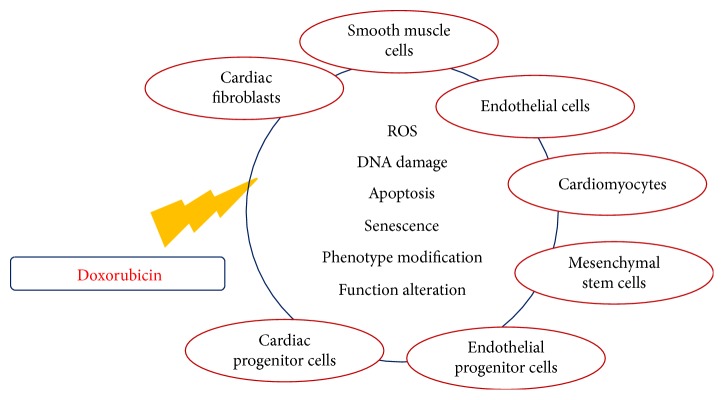
Harmful actions of doxorubicin. Simplified scheme of doxorubicin-targeted cell types and molecular and cellular effects. See text for details.

Cardiomyocytes account for less than one-third of the total number of cells within the heart. Therefore, it is reasonable that the disruption of cardiovascular homeostasis by DOX may also depend on other cellular components, especially when DOX cardiotoxicity is viewed as a chronic and progressive continuum that cumulates in a circulatory failure (Figure 1).

Given the “universal” role of ROS in physiological signalling and diseases, the importance of ROS hypothesis in the pathogenesis of DOX cardiomyopathy can be further emphasized when taking into consideration that smooth muscle cells, endothelial cells of the endocardium and coronary vessels, cardiac fibroblasts, and other interstitial cells together with a growing array of cells with progenitor characteristics are involved in cardiovascular homeostasis (Table 1).

## 4. Cardiac Progenitor Cells

The ability of self-regeneration, remarkable in foetal and neonatal mammalian hearts, reaches limited extent in the adult cardiac tissue. Despite this aspect, the presence of an endogenous compartment of amplifying cells in the adult heart has been repeatedly demonstrated [44–47]. The most extensively characterized pool of primitive cells is represented by cardiac progenitor cells (CPCs) and c-kit-positive and multipotent cells residing in the myocardium, whose contribution in tissue homeostasis/repair has been documented in several diseases, both in humans and rodent models [48–55]. Evidence from basic research studies have suggested an additional mechanism of DOX-induced cardiotoxicity, pointing to the effects exerted by DOX on the endogenous pool of CPCs [48, 56–60]. In an animal model of anthracycline cardiomyopathy, DOX increased ROS-induced DNA damage, cell cycle arrest, cellular senescence, and apoptosis, thus affecting CPC growth and functional properties. The depletion of the CPC pool in the myocardium interfered with mechanisms that account for the restoration of the structural and functional integrity of the failing heart [48]. Supporting evidence have come from a following study in which the hearts from oncologic patients, who died of heart failure after treatment with chemotherapeutic drugs, including anthracyclines, were analyzed. With respect to age-matched controls who died from noncardiovascular causes, the myocardium of DOX-treated patients showed a higher number of CPCs labelled with the phosphorylated form of histone H2AX and p16^INK4a^, indicating the accumulation of oxidative DNA damage and cellular senescence, respectively [56]. Moreover, human CPCs exposed to DOX *in vitro*, experienced the activation of senescent and proapoptotic pathways, corroborating the concept that a deficiency in CPC function may be responsible for a higher susceptibility of the myocardium to injury [56]. Indeed, after exposure to DOX, human CPCs could not induce any structural and functional recovery when injected in the heart of animals affected by DOX cardiomyopathy, confirming the ineffectiveness of DOX-exposed CPCs in fulfilling their functional role in the diseased myocardium [57]. Interestingly, the treatment with resveratrol, a sirtuin 1 activator with intrinsic antioxidant properties, was able to prevent senescence and growth arrest of CPCs, by decreasing intracellular ROS accumulation and enhancing oxidative stress defence. Moreover, myocardial delivery of CPCs primed with resveratrol partly restored cardiac function and significantly improved animal survival [57]. A recent study conducted on human CPCs confirmed the main role played by senescence and apoptosis in mediating deleterious effects of DOX. The frequency of senescence-associated-*β*-galactosidase (SA-*β*-gal) positive cells increased significantly after DOX, while pretreatment with human amniotic fluid stem cell secretome limited cell damage and protected CPCs against DOX [59].

### 4.1. CPCs and microRNAs

MicroRNAs (miRNAs or miRs) are emerging as new regulators of cardiovascular function given their contribution in modulating several biological processes including the response to oxidative stress and cell damage [61]. As noncoding RNAs, miRNAs have been reported to regulate cardiac cell proliferation and differentiation [62], and among others, the miR-34 family, and in particular miR-34a, is predominantly expressed in the heart where it is associated with DNA damage, senescence, and apoptosis in cardiac cells [63–65]. Recent evidence demonstrated the increased expression of miR-34a in rat CPCs after exposure to DOX [58]. miR-34a determined the activation of p16^INK4a^- and p53-mediated prosenescent and proapoptotic signalling in this cell population and when released by DOX-treated CPCs, affected viability and function of myocytes, fibroblasts, and endothelial cells [58]. The latter mode of action suggests a paracrine role as already shown in other cardiovascular pathologies [66–69]. However, the implications of miR-34a modulation in oncologic patients, as well as the hypothetical role as a biomarker of myocardial damage needs to be further evaluated.

### 4.2. CPCs and Late Cardiotoxicity

Despite decades of researches, there is no general agreement on the molecular mechanisms through which DOX, inducing cell abnormalities, produces late cardiotoxicity. A possible explanation is that exposure to DOX, even at a dosage not determining symptomatic manifestations of cardiotoxic events, makes the heart more susceptible to successive insults, with impaired angiogenesis, wound healing, or progenitor cell function. To test this hypothesis, juvenile mice were exposed to DOX with a cumulative dose not inducing acute cardiotoxicity, and once adults, they were subjected to myocardial infarction [60]. In comparison to infarcted mice treated with saline, DOX-exposed mice were more sensitive to myocardial infarction, with a greater extent of infarct size and fibrosis, and a more reduced blood vessel formation in the infarct border zone [60]. Moreover, DOX reduced the number of CPCs *in vivo* and affected their functional competence by inhibiting cell growth and differentiation capacity *in vitro*. The cell cycle inhibitor p16^INK4a^ was significantly upregulated in CPCs from hearts exposed to DOX, suggesting the involvement of cellular senescence progenitors as one of the mechanisms responsible of the higher susceptibility of the heart to stress [60]. In this scenario, CPCs “poisoned” by DOX fail to migrate to the site of injury with a consequent defect in myocardial repair. The major impact of senescence in affecting CPC behaviour was confirmed in an *in vitro* study, in which human CPCs were treated with DOX. After anthracycline washout, the fraction of p16^INK4a^ positive cells was significantly increased, indicating DOX-induced activation of the cellular senescent pathway and irreversible arrest of cell growth [56].

Although DOX clearly targets and affects CPC population, the direct link between DOX-induced CPC deterioration and alterations of myocardial structure and function is still to be defined. Altogether, these results support the hypothesis that an early toxic event may be responsible of an asymptomatic cardiomyopathy resulting in a late-onset heart failure.

## 5. Vascular Cells

In cancer biology, angiogenesis plays a major role given the necessity of new blood vessels needed by the tumour mass to support its own growth, and continuous researches have pointed the attention on the effects exerted by cytotoxic drugs upon the vascular system [70]. On the other hand, vascular damage can be directed on nontumour districts as well, supporting the notion that endothelial toxicity constitutes an additional aspect of antineoplastic therapies including anthracycline-related cardiovascular complications [11, 71, 72]. Studies conducted on endothelial cells demonstrated that increases in cytoplasmic ROS and DNA damage induced by anthracyclines determine detrimental responses in the activity of the endothelium and disruption of nitric oxide/superoxide balance [73–75]. It has been shown that DOX can bind to eNOS that reduces DOX to the semiquinone radical with a consequent increase in superoxide formation and a decrease in nitric oxide production [16]. In endothelial cells, DOX-induced apoptosis was linked to an elevation in intracellular calcium levels and paralleled by enhanced transcription of eNOS, suggesting a role for eNOS in DOX-mediated endothelial cell death [73]. Additionally, an appealing concept of cardiac microvascular injury as a potential primary event that contributes to DOX-induced cardiotoxicity has recently emerged. DOX can affect the function of cardiac endothelial cell barrier by affecting the formation of tight junctions thus determining an increased vascular permeability [76].

Besides a direct effect on skeletal muscle microcirculation [77], the treatment with DOX has also proven to affect smooth muscle cells (SMCs). SMCs exposed to DOX underwent cellular senescence and cell cycle arrest and experienced the common characteristics of senescent cells, such as DNA damage, generation of ROS, and SA-*β*-gal activity [78]. A decrease in vessel relaxation was observed in organ culture studies as confirmation of DOX toxicity on the vascular system. The involvement of oxidative stress was evidenced by the partial restoration of vessel contractility in presence of superoxide dismutase [79, 80]. These data further support the view of cardiotoxicity of DOX as a multicellular effects-driven process. This can stimulate future studies aimed at better characterization of the mechanisms of vascular toxicity of anthracyclines and help to design more effective intervention strategies to prevent or minimize the impact of vascular cell dysfunction. Significantly, given the importance in identifying subclinical cardiovascular damage and avoid later complications, the scientific community is promoting the routine assessment of vascular function in cancer patients.

## 6. Cardiac Fibroblasts

Cardiac fibroblasts have been underappreciated for a long time. These cells, however, are essential for maintaining normal cardiac function and take a vital part in cardiac remodelling during pathological conditions. Myocardial fibrosis is a common feature of a broad variety of cardiovascular pathologies including anthracycline cardiomyopathy [81, 82]. Initiation and maintenance of fibrogenic response are regulated by a complex interaction of growth factors and cytokines. In particular, transforming growth factor-*β* (TGF-*β*) and its downstream effectors trigger the activation of interstitial fibroblasts and their transformation in myofibroblasts thus inducing the formation of extracellular matrix components, such as collagen type I [82–86]. As signalling molecules driving cardiac fibrosis, ROS are believed to be involved in the amplification of TGF-*β*-related pathways that promote fibroblast differentiation via NADPH oxidase [87]. In a rat model of DOX cardiomyopathy, oxidative stress, as a profibrotic effector, was accompanied by the upregulation of TGF-*β*, connective tissue growth factor, and SMAD3 and determined adverse matrix remodelling with accumulation of collagen type I. Moreover, treatment with DOX promoted the phenotypic transformation of cardiac fibroblasts into myofibroblasts both *in vivo* and *in vitro* [88]. The population of cardiac fibroblasts can be particularly vulnerable because of being exposed to both stimulatory and inhibitory signals. In cardiovascular diseases, senescence is a well-recognized process that contributes to inflammation and myocardial fibrosis and stimulates the production of several factors including IL-6, IL-8, TGF-*β*, and tumor necrosis factor *α* (TNF*α*) [89–91]. As one of the cardiotoxicity-driving molecules, the latter may have a relevant role as a TNF*α* receptor upregulation after DOX exposure can favour apoptosis in myocardial cells [92, 93]. Similar to other cell types, DOX induces the DNA damage-response system also in fibroblasts, in parallel with an increase in *γ*-H2AX nuclear foci. The activation of a stress sensor ataxia telangiectasia-mutated (ATM) kinase, which in turn catalyzes the phosphorylation of p53 in Ser15, leading to increased levels of p53 and p21 and hypophosphorylation of the retinoblastoma protein. Of note, in addition to the activation of the DNA damage-response cascade of molecular events, DOX produces a prompt reduction in the levels of acetyl-CoA carboxylase 1, the enzyme that catalyzes the rate-limiting step in fatty acid synthesis. Such induction of synchronized inhibition of proliferation and anabolism by DOX was seen in pulmonary fibroblasts [94]. In a recent study, cardiac fibroblasts exposed to DOX prematurely acquired a senescent phenotype, too, as shown by the increases in SA-*β*-gal activity and the expression of senescence markers p16^INK4a^ and p21 [95]. Cardiac fibroblasts have been even proposed as the principal cells that mediate cardiotoxic effects of DOX. It has been shown that ATM, located and activated mainly in cardiac fibroblasts, promotes a release of Fas ligand from fibroblasts thus facilitating DOX-induced cardiomyocyte apoptosis [81]. Overall, these processes can regulate the equilibrium of the myocardium and contribute to the switch to a profibrotic profile. Further studies will need to determine the relative contribution of cardiac fibroblasts in the pathophysiology of anthracycline cardiomyopathy and establish the modality and significance of fibroblast-cardiomyocyte cross talk in drug-induced cardiotoxicity.

## 7. Mesenchymal Stem Cells

Many pieces of evidence have indicated nonresident progenitors as cells capable to promote the repair of the damaged myocardium, pointing at the bone marrow as the principal source of these cells. Mesenchymal stem cells (MSCs) derive from adult tissues and are identified as an adherent, fibroblast-like population, originally isolated from the bone marrow but present in other tissues such as the skeletal muscle, adipose tissue, umbilical cord, amniotic fluid, and lung. Although their ability to differentiate into cardiomyocytes and contribute to functional recovery has not been definitively proven, their participation in activating the local repair machinery in the injured myocardium has been repetitively reported [96–100].

From a technical aspect, the use of MSC-derived from sources other than the bone marrow, (e.g., adipose tissue) is relatively easy and reproducible making this cell population valuable for application in regenerative medicine. Of note, transplantation of adipose tissue-derived MSCs was associated with beneficial effects on heart function after experimental myocardial infarction [101, 102] and on the vascular system by promoting revascularization and tissue repair in a murine model of hind limb ischemia [103, 104]. It is evident that a paracrine mode of action represents the main mechanism through which MSCs stimulate tissue repair. MSCs are able to produce and secrete a broad variety of cytokines, chemokines, and growth factors serving as supportive signalling for other cells directly involved in the repair of the injured myocardium.

The bone marrow is among the tissues severely injured by DOX, which has detrimental effects on local stem cell compartment including bone marrow-derived MSCs. In the context of DOX-dependent increase in oxidative stress, several aspects of MSC biology can be taken into consideration. Although MSCs are equipped with efficient enzymatic and nonenzymatic antioxidant mechanisms [105], excess ROS can influence growth, self-renewal, and differentiation of MSCs [106, 107]. Moreover, when MSCs respond to ROS with a stress-induced premature senescence [108], this cellular process has consequences on cell secretome manifested as the acquisition of a specific, senescence-associated secretory phenotype [109], and the decreased ability to secrete trophic factors [110]. It is also possible that oxidative stress and senescence have an impact on anti-inflammatory and immunomodulatory properties of MSCs [111]. Indeed, MSCs isolated from animals subjected to DOX administration exhibited a lower proliferation rate and had a limitative capacity to respond to cardiomyogenic differentiation stimuli and when treated *in vitro* with sublethal dose of DOX, experienced premature senescence and reduced clonogenicity [112, 113].

## 8. Endothelial Progenitor Cells

The finding that endothelial progenitor cells (EPCs) can home to the site of injury and regulate local angiogenesis and vascular repair has boosted the interest in their potential use for therapeutic purposes [114]. Importantly, the maintenance of the cardiovascular system homeostasis requires an adequate number of functional EPCs. This concept is supported by the correlation between the number of circulating EPCs and cardiovascular events [115]. The capacity of EPCs to restore angiogenesis after a vascular insult is hampered by stress-induced cellular aging processes. DOX, as an agent inducing premature senescence, has been shown to affect EPC function by increasing oxidative stress and activating senescence pathways with the involvement of NADPH oxidase [116]. In addition, subapoptotic doses of DOX accelerated senescence of EPCs by regulating p38 and JNK mitogen-activated protein kinases and triggering p16^INK4a^-dependent signalling [117]. Therefore, ROS accumulation and induction of senescence seem to be key mechanisms implicated in the effects that DOX exerts on EPCs thus hindering their functional capacity.

## 9. Antioxidant Strategies as Cardioprotection

Given the abundant evidence pointing at oxidative stress as a key event in the pathophysiology of anthracycline cardiomyopathy, the use of several compounds with antioxidant property, coadministered with chemotherapeutic agents, has been explored to counteract the clinical manifestations of cardiotoxicity. Important work has been conducted *in vitro* and on animal models to evaluate the effectiveness of adding an antioxidant therapy to anthracycline regimens, in order to reduce oxidative damage upon cardiac cells. Early studies assessed the efficacy of dietary supplements such as vitamin A, vitamin C, vitamin E, coenzyme Q, omega-3 fatty acids, and flavonoids or other compounds known to prevent oxidative damage. Despite promising results based on preclinical studies, only a minority of these compounds has entered clinical trials and even less has shown a positive impact on heart function and structure [118–130]. A fundamental premise for a successful and safe use of adjuvants in association with chemotherapeutics consists in their capacity of not interfering with the cytotoxic effects of antineoplastic drugs. *In vitro* studies evidenced a “neutral” activity of most of these compounds in reducing antineoplastic efficacy of anthracyclines in several tumor cells, confirming the observation that the mechanisms involved in antitumor activity may differ from those affecting noncancer cells [131–134].

Some clinical trials, summarized below, tested the hypothesis that coadministration of antioxidants with cancer chemotherapeutic agents protects the heart, although data on the tumor response rate were largely missing.

N-Acetylcysteine has been shown to reduce biological oxidants by promoting intracellular glutathione synthesis. However, a randomized controlled trial evaluating the prevention of DOX cardiomyopathy with N-acetylcysteine reported no benefits since the rate of heart failure was similar between the groups [135].

Amifostine is a cytoprotective adjuvant against the effects of radiation and chemotherapy. Although the evidence of benefits in a rat model of DOX-induced cardiotoxicity in rats were shown [136], a randomized controlled trial conducted on paediatric patients treated with cisplatin and DOX showed no functional recovery of the heart when patients were additionally infused with amifostine [137].

Coenzyme Q10 is an intracellular antioxidant that protects the membrane phospholipids and proteins from free radical-induced oxidative damage. Results from a small trial enrolling paediatric cancer patients displayed beneficial effects on heart function in the group receiving oral coenzyme Q10 during chemotherapy [138]. However, these preliminary findings have never been verified in larger clinical trials.

Dexrazoxane, an intracellular iron-chelating agent, was found effectively cardioprotective against anthracycline-induced cardiotoxicity in several randomized trials in both children and adults [139–141]. Although it is the only cardioprotective drug approved in chemotherapy settings, concerns regarding the possible interaction with the antitumor efficacy of anthracyclines or the potential risk of a second malignancy in paediatric patients have limited its application. This implied a restraint of indications for clinical use by the Food and Drug Administration and European Medicine Agency, which is restricted to breast cancer patients receiving a cumulative dose that exceeds 300 mg/m^2^ DOX or 540 mg/m^2^ epirubicin [140–143]. Since no other iron chelator has shown cardioprotective effect [144], it is likely that iron chelating-dependent ROS formation does not predominantly represent the mechanism of action of dexrazoxane. Indeed, the inhibition of Top2-*β* that prevents DNA double strand breaks and cell death seems to be the most accredited mechanism [145], confirmed by experimental findings claiming no cardioprotection of dexrazoxane derivatives lacking activity on Top2-*β* [146].

Two *β*-adrenergic blocking agents, carvedilol and nebivolol, have been reported to improve cardiac function in patients treated with DOX [147, 148]. A small clinical trial on the prophylactic use of carvedilol in patients undergoing anthracycline chemotherapy showed reduced incidence of systolic and diastolic dysfunction in the group treated with a combination of DOX and oral carvedilol [147]. Moreover, clinical evidence of the cardioprotective effect of carvedilol have come from a study conducted in children with acute lymphoblastic leukaemia treated with DOX, in which carvedilol pretreatment of DOX-treated patients resulted in a significant increase in systolic function and decrease of troponin I levels [149]. Nebivolol had a protective effect on heart function in comparison to placebo, in patients with breast cancer receiving DOX [148]. According to a consensus based on experimental data, the cardioprotective activity of these drugs is not to be ascribed to their properties as *β*-blockers but rather depends on alternative mechanisms. Carvedilol, displaying antioxidant properties, is able to prevent lipid peroxidation and the depletion of endogenous scavengers [123]. On the other hand, the success of the treatment with nebivolol is linked to nitric oxide-dependent vasodilation ability and the prevention of peroxynitrite accumulation [150]. Interestingly, the association of a *β*-blocker (carvedilol) with the ACE-inhibitor (enalapril) provided the most effective response toward the normalization of anthracycline-caused decrease in ejection fraction, when either administered preventively, without any signs of systolic dysfunction [151], or promptly given after detection of ejection fraction impairment [152].

The potential role of statins in anthracycline cardiotoxicity has been associated with pleiotropic effects, exerted as anti-inflammatory and antioxidant actions [153]. In particular, statins enhanced antioxidant defence and mitigated cardiac inflammation following DOX treatment [154, 155]. In the clinical setting, the use of statins in chemotherapy-receiving cancer patients was associated with a preservation of heart function and a reduced risk of heart failure and cardiac-related mortality. In two small trials conducted on patients undergoing anthracycline-based chemotherapy, the group cotreated with statins (atorvastatin or simvastatin) experienced no change in systolic function, whereas the group not receiving the statin showed a significant decrease in ejection fraction [156–158].

For all the compounds discussed above, and in particular for those that have shown a cardioprotective profile, there is a need of additional studies recruiting a higher number of patients that may provide stronger evidence of the cardioprotective effect.

The studies of antioxidants in cardiovascular diseases need to consider that in living organisms, high concentration ROS elicit cellular damage, while at lower concentrations they act as signalling molecules regulating many cellular responses [159–164]. Such a dual profile that makes ROS play opposite roles in the cellular signalling networks according to physiological and pathological conditions may have a significant consequence on the outcomes of the interventions aimed at redox status of a cell. Indeed, in spite of the strong rationale for therapeutic targeting of redox pathways in anthracycline cardiomyopathy, the antioxidant therapy has failed to give a substantial contribution in terms of cardioprotection. Another aspect that makes the separation of beneficial outcomes even more challenging is that antioxidants themselves may show a two-faced nature, acting concurrently as antiradicals and prooxidants. Indeed, while the use of antioxidants is increasing, this kind of supplementation is considered unhelpful at best [165]. Nutritional supplements marketed for the prevention of cardiovascular diseases are not specifically screened for their effects in different physiological and pathological conditions of the cardiovascular system. Better understanding of redox mechanisms controlling a variety of reactive oxygen metabolites in cardiovascular pathophysiology is likely to allow the design of new studies for the use of antioxidants in cardiac diseases [159].

The notion that subcellular compartmentalization of ROS and ROS-mediated signalling can be of vital importance for both cardiovascular physiology and response to stressors [160] should influence the development of new intervention strategies. In this regard, targeting antioxidants to specific compartments, in order to interfere with a defined subcellular pathological signalling, could favour the expected effects over the location-unspecific and possibly undesirable action of antioxidant agents that have been tried in the past.

## 10. Conclusion

DOX cardiotoxicity is becoming an interdisciplinary point of interest that in its clinical aspect asks for the closer cooperation between cardiologists, oncologists, and pharmacologists to develop new therapeutic strategies aiming at reducing or preventing early and late cardiotoxic events. However, basic research efforts by molecular and cellular biology teams will be necessary to advance our understanding of this relatively old but still troubling problem.

## Figures and Tables

**Table 1:** Doxorubicin-induced toxic effects in cardiac and extracardiac cells.

Cell type	Study	Mechanism of toxicity
CPCs	De Angelis et al. [48]	Oxidative stress, cell cycle arrest, and apoptosis
CPCs	Piegari et al. [56]	DNA damage and senescence
CPCs	De Angelis et al. [57]	Oxidative stress, senescence, apoptosis, and reduced migratory capacity
CPCs	Piegari et al. [58]	Senescence and apoptosis
CPCs	Lazzarini et al. [59]	Senescence and apoptosis
CPCs	Huang et al. [60]	Senescence and impaired differentiation potential
ECs	Kalivendi et al. [73]	Oxidative stress, NO deficit, and apoptosis
ECs	Kotamraju et al. [74]	Oxidative stress and apoptosis
ECs	Wojcik et al. [72]	Oxidative stress, apoptosis, and reduced elasticity
ECs	Wilkinson et al. [76]	Impaired microvascular permeability
SMCs	Bielak-Zmijewska et al. [78]	Oxidative stress and senescence
SMCs	Murata et al. [79]	DNA damage and apoptosis
SMCs	Murata et al. [80]	Impaired relaxation and apoptosis
CFs	Zhan et al. [81]	Apoptosis
CFs	Cappetta et al. [88]	Profibrotic phenotype
CFs	Marmisolle et al. [94]	Senescence
CFs	Ghosh et al. [95]	Senescence
MSCs	Yang et al. [110]	Apoptosis and decreased secretory function
MSCs	Oliveira et al. [112]	Lower proliferation rate and impaired differentiation potential
MSCs	Buttiglieri et al. [113]	Telomere length shortening and reduced clonogenic potential
EPCs	De Falco et al. [116]	Oxidative stress and senescence
EPCs	Spallarossa et al. [117]	Senescence and reduced migratory capacity
EPCs	Yasuda et al. (2010)	Senescence and defective engraftment

CPCs: cardiac progenitor cells; ECs: endothelial cells; SMCs: smooth muscle cells; CFs: cardiac fibroblasts; MSCs: mesenchymal stem cells; EPCs: endothelial progenitor cells.

## References

[1] Howlader N., Noone A. M., Krapcho M. *SEER Cancer Statistics Review, 1975-2014*.

[2] Zamorano J. L., Lancellotti P., Rodriguez Muñoz D. (2017). 2016 ESC Position Paper on cancer treatments and cardiovascular toxicity developed under the auspices of the ESC Committee for Practice Guidelines. *European Journal of Heart Failure*.

[3] Armenian S. H., Lacchetti C., Barac A. (2017). Prevention and monitoring of cardiac dysfunction in survivors of adult cancers: American Society of Clinical Oncology Clinical Practice Guideline. *Journal of Clinical Oncology*.

[4] Farías J. G., Molina V. M., Carrasco R. A. (2017). Antioxidant therapeutic strategies for cardiovascular conditions associated with oxidative stress. *Nutrients*.

[5] Ewer M. S., Ewer S. M. (2010). Troponin I provides insight into cardiotoxicity and the anthracycline-trastuzumab interaction. *Journal of Clinical Oncology*.

[6] Deidda M., Madonna R., Mango R. (2016). Novel insights in pathophysiology of antiblastic drugs-induced cardiotoxicity and cardioprotection. *Journal of Cardiovascular Medicine*.

[7] Minotti G., Menna P., Salvatorelli E., Cairo G., Gianni L. (2004). Anthracyclines: molecular advances and pharmacologic developments in antitumor activity and cardiotoxicity. *Pharmacological Reviews*.

[8] Takemura G., Fujiwara H. (2007). Doxorubicin-induced cardiomyopathy from the cardiotoxic mechanisms to management. *Progress in Cardiovascular Diseases*.

[9] Nithipongvanitch R., Ittarat W., Cole M. P., Tangpong J., Clair D. K., Oberley T. D. (2007). Mitochondrial and nuclear p53 localization in cardiomyocytes: redox modulation by doxorubicin (Adriamycin)?. *Antioxidants & Redox Signaling*.

[10] Gianni L., Herman E. H., Lipshultz S. E., Minotti G., Sarvazyan N., Sawyer D. B. (2008). Anthracycline cardiotoxicity: from bench to bedside. *Journal of Clinical Oncology*.

[11] Tocchetti C. G., Cadeddu C., Di Lisi D. (2017). From molecular mechanisms to clinical management of antineoplastic drug-induced cardiovascular toxicity: a translational overview. *Antioxidants & Redox Signaling*.

[12] Stěrba M., Popelová O., Vávrová A. (2013). Oxidative stress, redox signaling, and metal chelation in anthracycline cardiotoxicity and pharmacological cardioprotection. *Antioxidants & Redox Signaling*.

[13] Angsutararux P., Luanpitpong S., Issaragrisil S. (2015). Chemotherapy-induced cardiotoxicity: overview of the roles of oxidative stress. *Oxidative Medicine and Cellular Longevity*.

[14] Schlame M., Rua D., Greenberg M. L. (2000). The biosynthesis and functional role of cardiolipin. *Progress in Lipid Research*.

[15] Pani G., Bedogni B., Anzevino R. (2000). Deregulated manganese superoxide dismutase expression and resistance to oxidative injury in p53-deficient cells. *Cancer Research*.

[16] Vásquez-Vivar J., Martasek P., Hogg N., Masters B. S., Pritchard K. A., Kalyanaraman B. (1997). Endothelial nitric oxide synthase-dependent superoxide generation from Adriamycin. *Biochemistry*.

[17] Liu B., Li H., Qu H., Sun B. (2006). Nitric oxide synthase expressions in ADR-induced cardiomyopathy in rats. *Journal of Biochemistry and Molecular Biology*.

[18] Neilan T. G., Blake S. L., Ichinose F. (2007). Disruption of nitric oxide synthase 3 protects against the cardiac injury, dysfunction, and mortality induced by doxorubicin. *Circulation*.

[19] Cole M. P., Chaiswing L., Oberley T. D. (2006). The protective roles of nitric oxide and superoxide dismutase in adriamycin-induced cardiotoxicity. *Cardiovascular Research*.

[20] Mukhopadhyay P., Rajesh M., Bátkai S. (2009). Role of superoxide, nitric oxide, and peroxynitrite in doxorubicin-induced cell death in vivo and in vitro. *American Journal of Physiology Heart and Circulatory Physiology*.

[21] Zhang M., Perino A., Ghigo A., Hirsch E., Shah A. M. (2013). NADPH oxidases in heart failure: poachers or gamekeepers?. *Antioxidants & Redox Signaling*.

[22] Gilleron M., Marechal X., Montaigne D., Franczak J., Neviere R., Lancel S. (2009). NADPH oxidases participate to doxorubicin-induced cardiac myocyte apoptosis. *Biochemical and Biophysical Research Communications*.

[23] Pacher P., Liaudet L., Bai P. (2003). Potent metalloporphyrin peroxynitrite decomposition catalyst protects against the development of doxorubicin-induced cardiac dysfunction. *Circulation*.

[24] Cappetta D., Esposito G., Coppini R. (2017). Effects of ranolazine in a model of doxorubicin-induced left ventricle diastolic dysfunction. *British Journal of Pharmacology*.

[25] Zhao Y., McLaughlin D., Robinson E. (2010). Nox2 NADPH oxidase promotes pathologic cardiac remodeling associated with doxorubicin chemotherapy. *Cancer Research*.

[26] Reichwagen A., Ziepert M., Kreuz M. (2015). Association of NADPH oxidase polymorphisms with anthracycline-induced cardiotoxicity in the RICOVER-60 trial of patients with aggressive CD20^+^ B-cell lymphoma. *Pharmacogenomics*.

[27] Wojnowski L., Kulle B., Schirmer M. (2005). NAD(P)H oxidase and multidrug resistance protein genetic polymorphisms are associated with doxorubicin-induced cardiotoxicity. *Circulation*.

[28] Ghigo A., Li M., Hirsch E. (2016). New signal transduction paradigms in anthracycline-induced cardiotoxicity. *Biochimica et Biophysica Acta (BBA) - Molecular Cell Research*.

[29] McGowan J. V., Chung R., Maulik A., Piotrowska I., Walker J. M., Yellon D. M. (2017). Anthracycline chemotherapy and cardiotoxicity. *Cardiovascular Drugs and Therapy*.

[30] Zhang S., Liu X., Bawa-Khalfe T. (2012). Identification of the molecular basis of doxorubicin-induced cardiotoxicity. *Nature Medicine*.

[31] Matt S., Hofmann T. G. (2016). The DNA damage-induced cell death response: a roadmap to kill cancer cells. *Cellular and Molecular Life Sciences*.

[32] Pilco-Ferreto N., Calaf G. M. (2016). Influence of doxorubicin on apoptosis and oxidative stress in breast cancer cell lines. *International Journal of Oncology*.

[33] Shin H. J., Kwon H. K., Lee J. H. (2015). Doxorubicin-induced necrosis is mediated by poly-(ADP-ribose) polymerase 1 (PARP1) but is independent of p53. *Scientific Reports*.

[34] Spina A., Sorvillo L., Di Maiolo F. (2013). Inorganic phosphate enhances sensitivity of human osteosarcoma U2OS cells to doxorubicin via a p53-dependent pathway. *Journal of Cellular Physiology*.

[35] Sapio L., Sorvillo L., Illiano M., Chiosi E., Spina A., Naviglio S. (2015). Inorganic phosphate prevents Erk1/2 and Stat3 activation and improves sensitivity to doxorubicin of MDA-MB-231 breast cancer cells. *Molecules*.

[36] Brown S. A., Sandhu N., Herrmann J. (2015). Systems biology approaches to adverse drug effects: the example of cardio-oncology. *Nature Reviews Clinical Oncology*.

[37] Kankeu C., Clarke K., Passante E., Huber H. J. (2016). Doxorubicin-induced chronic dilated cardiomyopathy-the apoptosis hypothesis revisited. *Journal of Molecular Medicine*.

[38] Mazevet M., Moulin M., Llach-Martinez A. (2013). Complications of chemotherapy, a basic science update. *La Presse Médicale*.

[39] Childs B. G., Durik M., Baker D. J., van Deursen J. M. (2015). Cellular senescence in aging and age-related disease: from mechanisms to therapy. *Nature Medicine*.

[40] Chen Q. M., Tu V. C., Liu J. (2000). Measurements of hydrogen peroxide induced premature senescence: senescence-associated *β*-galactosidase and DNA synthesis index in human diploid fibroblasts with down-regulated p53 or Rb. *Biogerontology*.

[41] Rebbaa A., Zheng X., Chou P. M., Mirkin B. L. (2003). Caspase inhibition switches doxorubicin-induced apoptosis to senescence. *Oncogene*.

[42] Oh H., Schneider M. D. (2002). The emerging role of telomerase in cardiac muscle cell growth and survival. *Journal of Molecular and Cellular Cardiology*.

[43] Maejima Y., Adachi S., Ito H., Hirao K., Isobe M. (2008). Induction of premature senescence in cardiomyocytes by doxorubicin as a novel mechanism of myocardial damage. *Aging Cell*.

[44] Beltrami A. P., Barlucchi L., Torella D. (2003). Adult cardiac stem cells are multipotent and support myocardial regeneration. *Cell*.

[45] Smith R. R., Barile L., Cho H. C. (2007). Regenerative potential of cardiosphere-derived cells expanded from percutaneous endomyocardial biopsy specimens. *Circulation*.

[46] Bearzi C., Rota M., Hosoda T. (2007). Human cardiac stem cells. *Proceedings of the National Academy of Sciences of the United States of America*.

[47] Pfister O., Mouquet F., Jain M. (2005). CD31^−^ but not CD31^+^ cardiac side population cells exhibit functional cardiomyogenic differentiation. *Circulation Research*.

[48] De Angelis A., Piegari E., Cappetta D. (2010). Anthracycline cardiomyopathy is mediated by depletion of the cardiac stem cell pool and is rescued by restoration of progenitor cell function. *Circulation*.

[49] Urbanek K., Torella D., Sheikh F. (2005). Myocardial regeneration by activation of multipotent cardiac stem cells in ischemic heart failure. *Proceedings of the National Academy of Sciences of the United States of America*.

[50] Rota M., LeCapitaine N., Hosoda T. (2006). Diabetes promotes cardiac stem cell aging and heart failure, which are prevented by deletion of the p66shc gene. *Circulation Research*.

[51] Gonzalez A., Rota M., Nurzynska D. (2008). Activation of cardiac progenitor cells reverses the failing heart senescent phenotype and prolongs lifespan. *Circulation Research*.

[52] Avolio E., Gianfranceschi G., Cesselli D. (2014). Ex vivo molecular rejuvenation improves the therapeutic activity of senescent human cardiac stem cells in a mouse model of myocardial infarction. *Stem Cells*.

[53] Rupp S., Bauer J., von Gerlach S. (2012). Pressure overload leads to an increase of cardiac resident stem cells. *Basic Research in Cardiology*.

[54] D'Amario D., Leone A. M., Narducci M. L. (2017). Human cardiac progenitor cells with regenerative potential can be isolated and characterized from 3D-electro-anatomic guided endomyocardial biopsies. *International Journal of Cardiology*.

[55] Piegari E., Di Salvo G., Castaldi B. (2008). Myocardial strain analysis in a doxorubicin-induced cardiomyopathy model. *Ultrasound in Medicine & Biology*.

[56] Piegari E., De Angelis A., Cappetta D. (2013). Doxorubicin induces senescence and impairs function of human cardiac progenitor cells. *Basic Research in Cardiology*.

[57] De Angelis A., Piegari E., Cappetta D. (2015). SIRT1 activation rescues doxorubicin-induced loss of functional competence of human cardiac progenitor cells. *International Journal of Cardiology*.

[58] Piegari E., Russo R., Cappetta D. (2016). MicroRNA-34a regulates doxorubicin-induced cardiotoxicity in rat. *Oncotarget*.

[59] Lazzarini E., Balbi C., Altieri P. (2016). The human amniotic fluid stem cell secretome effectively counteracts doxorubicin-induced cardiotoxicity. *Scientific Reports*.

[60] Huang C., Zhang X., Ramil J. M. (2010). Juvenile exposure to anthracyclines impairs cardiac progenitor cell function and vascularization resulting in greater susceptibility to stress-induced myocardial injury in adult mice. *Circulation*.

[61] Mendell J. T., Olson E. N. (2012). MicroRNAs in stress signaling and human disease. *Cell*.

[62] Tao L., Bei Y., Zhou Y., Xiao J., Li X. (2015). Non-coding RNAs in cardiac regeneration. *Oncotarget*.

[63] Boon R. A., Iekushi K., Lechner S. (2013). MicroRNA-34a regulates cardiac ageing and function. *Nature*.

[64] Li N., Wang K., Li P. F. (2015). MicroRNA-34 family and its role in cardiovascular disease. *Critical Reviews in Eukaryotic Gene Expression*.

[65] Chen F., Hu S. J. (2012). Effect of microRNA-34a in cell cycle, differentiation, and apoptosis: a review. *Journal of Biochemical and Molecular Toxicology*.

[66] Matsumoto S., Sakata Y., Suna S. (2013). Circulating p53-responsive microRNAs are predictive indicators of heart failure after acute myocardial infarction. *Circulation Research*.

[67] Barile L., Lionetti V., Cervio E. (2014). Extracellular vesicles from human cardiac progenitor cells inhibit cardiomyocyte apoptosis and improve cardiac function after myocardial infarction. *Cardiovascular Research*.

[68] Viereck J., Bang C., Foinquinos A., Thum T. (2014). Regulatory RNAs and paracrine networks in the heart. *Cardiovascular Research*.

[69] Hergenreider E., Heydt S., Tréguer K. (2012). Atheroprotective communication between endothelial cells and smooth muscle cells through miRNAs. *Nature Cell Biology*.

[70] Soultati A., Mountzios G., Avgerinou C. (2012). Endothelial vascular toxicity from chemotherapeutic agents: preclinical evidence and clinical implications. *Cancer Treatment Reviews*.

[71] Di Lisi D., Madonna R., Zito C. (2017). Anticancer therapy-induced vascular toxicity: VEGF inhibition and beyond. *International Journal of Cardiology*.

[72] Wojcik T., Szczesny E., Chlopicki S. (2015). Detrimental effects of chemotherapeutics and other drugs on the endothelium: a call for endothelial toxicity profiling. *Pharmacological Reports*.

[73] Kalivendi S. V., Kotamraju S., Zhao H., Joseph J., Kalyanaraman B. (2001). Doxorubicin-induced apoptosis is associated with increased transcription of endothelial nitric-oxide synthase. *The Journal of Biological Chemistry*.

[74] Kotamraju S., Koronev E. A., Joseph J., Kalyanaraman B. (2000). Doxorubicin-induced apoptosis in endothelial cells and cardiomyocytes is ameliorated by nitrone spin traps and ebselen. *The Journal of Biological Chemistry*.

[75] Wojcik T., Buczek E., Majzner K. (2015). Comparative endothelial profiling of doxorubicin and daunorubicin in cultured endothelial cells. *Toxicology In Vitro*.

[76] Wilkinson E. L., Sidaway J. E., Cross M. J. (2016). Cardiotoxic drugs Herceptin and doxorubicin inhibit cardiac microvascular endothelial cell barrier formation resulting in increased drug permeability. *Biology Open*.

[77] Lübbe A. S. (1993). Doxorubicin and local hyperthermia in the microcirculation of skeletal muscle. *Cancer Chemotherapy and Pharmacology*.

[78] Bielak-Zmijewska A., Wnuk M., Przybylska D. (2014). A comparison of replicative senescence and doxorubicin-induced premature senescence of vascular smooth muscle cells isolated from human aorta. *Biogerontology*.

[79] Murata T., Yamawaki H., Hori M., Sato K., Ozaki H., Karaki H. (2001). Chronic vascular toxicity of doxorubicin in an organ-cultured artery. *British Journal of Pharmacology*.

[80] Murata T., Yamawaki H., Yoshimoto R. (2001). Chronic effect of doxorubicin on vascular endothelium assessed by organ culture study. *Life Sciences*.

[81] Zhan H., Aizawa K., Sun J. (2016). Ataxia telangiectasia mutated in cardiac fibroblasts regulates doxorubicin-induced cardiotoxicity. *Cardiovascular Research*.

[82] Leask A. (2015). Getting to the heart of the matter. *Circulation Research*.

[83] Krstić J., Trivanović D., Mojsilović S., Santibanez J. F. (2015). Transforming growth factor-beta and oxidative stress interplay: implications in tumorigenesis and cancer progression. *Oxidative Medicine and Cellular Longevity*.

[84] Li A. H., Liu P. P., Villarreal F. J., Garcia R. A. (2014). Dynamic changes in myocardial matrix and relevance to disease: translational perspectives. *Circulation Research*.

[85] Webb C. S., Bonnema D. D., Ahmed S. H. (2006). Specific temporal profile of matrix metalloproteinase release occurs in patients after myocardial infarction: relation to left ventricular remodelling. *Circulation*.

[86] Kuwahara F., Kai H., Tokuda K. (2002). Transforming growth factor-β function blocking prevents myocardial fibrosis and diastolic dysfunction in pressure-overloaded rats. *Circulation*.

[87] Cucoranu I., Clempus R., Dikalova A. (2005). NAD(P)H oxidase 4 mediates transforming growth factor-β1-induced differentiation of cardiac fibroblasts into myofibroblasts. *Circulation Research*.

[88] Cappetta D., Esposito G., Piegari E. (2016). SIRT1 activation attenuates diastolic dysfunction by reducing cardiac fibrosis in a model of anthracycline cardiomyopathy. *International Journal of Cardiology*.

[89] Zhu F., Li Y., Zhang J. (2013). Senescent cardiac fibroblast is critical for cardiac fibrosis after myocardial infarction. *PLoS One*.

[90] Muñoz-Espín D., Serrano M. (2014). Cellular senescence: from physiology to pathology. *Nature Reviews Molecular Cell Biology*.

[91] Ren J. L., Pan J. S., Lu Y. P., Sun P., Han J. (2009). Inflammatory signaling and cellular senescence. *Cellular Signalling*.

[92] Zhao L., Zhang B. (2017). Doxorubicin induces cardiotoxicity through upregulation of death receptors mediated apoptosis in cardiomyocytes. *Scientific Reports*.

[93] Chiosi E., Spina A., Sorrentino A. (2007). Change in TNF-*α* receptor expression is a relevant event in doxorubicin-induced H9c2 cardiomyocyte cell death. *Journal of Interferon & Cytokine Research*.

[94] Marmisolle I., Martínez J., Liu J. (2017). Reciprocal regulation of acetyl-CoA carboxylase 1 and senescence in human fibroblasts involves oxidant mediated p38 MAPK activation. *Archives of Biochemistry and Biophysics*.

[95] Ghosh A. K., Rai R., Park K. E. (2016). A small molecule inhibitor of PAI-1 protects against doxorubicin-induced cellular senescence: molecular basis. *Oncotarget*.

[96] Danieli P., Malpasso G., Ciuffreda M. C. (2015). Conditioned medium from human amniotic mesenchymal stromal cells limits infarct size and enhances angiogenesis. *Stem Cells Translational Medicine*.

[97] Gnecchi M., Zhang Z., Ni A., Dzau V. J. (2008). Paracrine mechanisms in adult stem cell signaling and therapy. *Circulation Research*.

[98] Caplan A. I., Dennis J. E. (2006). Mesenchymal stem cells as trophic mediators. *Journal of Cellular Biochemistry*.

[99] Pittenger M. F., Martin B. J. (2004). Mesenchymal stem cells and their potential as cardiac therapeutics. *Circulation Research*.

[100] Nagaya N., Fujii T., Iwase T. (2004). Intravenous administration of mesenchymal stem cells improves cardiac function in rats with acute myocardial infarction through angiogenesis and myogenesis. *American Journal of Physiology Heart and Circulatory Physiology*.

[101] Mazo M., Planat-Bénard V., Abizanda G. (2008). Transplantation of adipose derived stromal cells is associated with functional improvement in a rat model of chronic myocardial infarction. *European Journal of Heart Failure*.

[102] Madonna R., Petrov L., Teberino M. A. (2015). Transplantation of adipose tissue mesenchymal cells conjugated with VEGF-releasing microcarriers promotes repair in murine myocardial infarction. *Cardiovascular Research*.

[103] Bortolotti F., Ukovich L., Razban V. (2015). In vivo therapeutic potential of mesenchymal stromal cells depends on the source and the isolation procedure. *Stem Cell Reports*.

[104] Madonna R., Taylor D. A., Geng Y. J. (2013). Transplantation of mesenchymal cells rejuvenated by the overexpression of telomerase and myocardin promotes revascularization and tissue repair in a murine model of hindlimb ischemia. *Circulation Research*.

[105] Valle-Prieto A., Conget P. A. (2010). Human mesenchymal stem cells efficiently manage oxidative stress. *Stem Cells and Development*.

[106] Ko E., Lee K. Y., Hwang D. S. (2012). Human umbilical cord blood-derived mesenchymal stem cells undergo cellular senescence in response to oxidative stress. *Stem Cells and Development*.

[107] Alves H., Munoz-Najar U., De Wit J. (2010). A link between the accumulation of DNA damage and loss of multi-potency of human mesenchymal stromal cells. *Journal of Cellular and Molecular Medicine*.

[108] Kim J. S., Kim E. J., Kim H. J. (2011). Proteomic and metabolomic analysis of H2O2-induced premature senescent human mesenchymal stem cells. *Experimental Gerontology*.

[109] Coppe J. P., Desprez P. Y., Krtolica A., Campisi J. (2010). The senescence-associated secretory phenotype: the dark side of tumor suppression. *Annual Review of Pathology*.

[110] Yang F., Chen H., Liu Y. (2013). Doxorubicin caused apoptosis of mesenchymal stem cells via p38, JNK and p53 pathway. *Cellular Physiology and Biochemistry*.

[111] Sepulveda J. C., Tome M., Fernandez M. E. (2014). Cell senescence abrogates the therapeutic potential of human mesenchymal stem cells in the lethal endotoxemia model. *Stem Cells*.

[112] Oliveira M. S., Carvalho J. L., Campos A. C., Gomes D. A., de Goes A. M., Melo M. M. (2014). Doxorubicin has *in vivo* toxicological effects on *ex vivo* cultured mesenchymal stem cells. *Toxicology Letters*.

[113] Buttiglieri S., Ruella M., Risso A. (2011). The aging effect of chemotherapy on cultured human mesenchymal stem cells. *Experimental Hematology*.

[114] Urbich C., Dimmeler S. (2004). Endothelial progenitor cells: characterization and role in vascular biology. *Circulation Research*.

[115] Schmidt-Lucke C., Rössig L., Fichtlscherer S. (2005). Reduced number of circulating endothelial progenitor cells predicts future cardiovascular events: proof of concept for the clinical importance of endogenous vascular repair. *Circulation*.

[116] De Falco E., Carnevale R., Pagano F. (2016). Role of NOX2 in mediating doxorubicin-induced senescence in human endothelial progenitor cells. *Mechanisms of Ageing and Development*.

[117] Spallarossa P., Altieri P., Barisione C. (2010). p38 MAPK and JNK antagonistically control senescence and cytoplasmic p16INK4A expression in doxorubicin-treated endothelial progenitor cells. *PLoS One*.

[118] Buyukokuroglu M. E., Taysi S., Buyukavci M., Bakan E. (2004). Prevention of acute adriamycin cardiotoxicity by dantrolene in rats. *Human & Experimental Toxicology*.

[119] Husken B. C., de Jong J., Beekman B., Onderwater R. C., van der Vijgh W. J., Bast A. (1995). Modulation of the in vitro cardiotoxicity of doxorubicin by flavonoids. *Cancer Chemotherapy and Pharmacology*.

[120] Iliskovic N., Hasinoff B. B., Malisza K. L., Li T., Danelisen I., Singal P. K. (1999). Mechanism of beneficial effects of probucol in adriamycin cardiomyopathy. *Molecular and Cellular Biochemistry*.

[121] Kumar D., Kirshenbaum L. A., Li T., Danelisen I., Singal P. K. (2001). Apoptosis in adriamycin cardiomyopathy and its modulation by probucol. *Antioxidants & Redox Signaling*.

[122] Lubawy W. C., Whaley J., Hurley L. H. (1979). Coenzyme Q10 or alpha-tocopherol reduces the acute toxicity of anthramycin in mice. *Research Communications in Chemical Pathology and Pharmacology*.

[123] Matsui H., Morishima I., Numaguchi Y., Toki Y., Okumura K., Hayakawa T. (1999). Protective effects of carvedilol against doxorubicin-induced cardiomyopathy in rats. *Life Sciences*.

[124] Milei J., Boveris A., Llesuy S. (1986). Amelioration of adriamycin-induced cardiotoxicity in rabbits by prenylamine and vitamins A and E. *American Heart Journal*.

[125] Oliveira P. J., Bjork J. A., Santos M. S. (2004). Carvedilol-mediated antioxidant protection against doxorubicin-induced cardiac mitochondrial toxicity. *Toxicology and Applied Pharmacology*.

[126] Shimpo K., Nagatsu T., Yamada K. (1991). Ascorbic acid and adriamycin toxicity. *The American Journal of Clinical Nutrition*.

[127] Tesoriere L., Ciaccio M., Valenza M. (1994). Effect of vitamin A administration on resistance of rat-heart against doxorubicin-induced cardiotoxicity and lethality. *The Journal of Pharmacology and Experimental Therapeutics*.

[128] van Acker S. A., Boven E., Kuiper K. (1997). Monohydroxyethylrutoside, a dose-dependent cardioprotective agent, does not affect the antitumor activity of doxorubicin. *Clinical Cancer Research*.

[129] Ojha S., Al Taee H., Goyal S. (2016). Cardioprotective potentials of plant-derived small molecules against doxorubicin associated cardiotoxicity. *Oxidative Medicine and Cellular Longevity*.

[130] Teng L. L., Shao L., Zhao Y. T., Yu X., Zhang D. F., Zhang H. (2010). The beneficial effect of *n*-3 polyunsaturated fatty acids on doxorubicin-induced chronic heart failure in rats. *The Journal of International Medical Research*.

[131] Cervantes A., Pinedo H. M., Lankelma J., Schuurhuis C. J. (1988). The role of oxygen-derived free radicals in the cytotoxicity of doxorubicin in multidrug resistant and sensitive human ovarian cancer cells. *Cancer Letters*.

[132] Myers C. E., McGuire W. P., Liss R. H., Ifrim I., Grotzinger K., Young R. C. (1977). Adriamycin: the role of lipid peroxidation in cardiac toxicity and tumor response. *Science*.

[133] Woodman R. J., Cysyk R., Kline I., Gang M., Venditti J. M. (1975). Enhancement of the effectiveness of daunorubicin (NSC-82151) or adriamycin (NSC-123127) against early mouse l1210 leukemia with ICRF-159 (NSC-129943). *Cancer Chemotherapy Reports*.

[134] Yoda Y., Nakazawa M., Abe T., Kawakami Z. (1986). Prevention of doxorubicin myocardial toxicity in mice by reduced glutathione. *Cancer Research*.

[135] Myers C., Bonow R., Palmeri S. (1983). A randomized controlled trial assessing the prevention of doxorubicin cardiomyopathy by N-acetylcysteine. *Seminars in Oncology*.

[136] Dragojevic-Simic V. M., Dobric S. L., Bokonjic D. R. (2004). Amifostine protection against doxorubicin cardiotoxicity in rats. *Anti-Cancer Drugs*.

[137] Gallegos-Castorena S., Martínez-Avalos A., Mohar-Betancourt A., Guerrero-Avendaño G., Zapata-Tarrés M., Medina-Sansón A. (2007). Toxicity prevention with amifostine in pediatric osteosarcoma patients treated with cisplatin and doxorubicin. *Pediatric Hematology and Oncology*.

[138] Iarussi D., Auricchio U., Agretto A. (1994). Protective effect of coenzyme Q10 on anthracyclines cardiotoxicity: control study in children with acute lymphoblastic leukemia and non-Hodgkin lymphoma. *Molecular Aspects of Medicine*.

[139] Speyer J. L., Green M. D., Zeleniuch-Jacquotte A. (1992). ICRF-187 permits longer treatment with doxorubicin in women with breast cancer. *Journal of Clinical Oncology*.

[140] Lipshultz S. E., Scully R. E., Lipsitz S. R. (2010). Assessment of dexrazoxane as a cardioprotectant in doxorubicin-treated children with high-risk acute lymphoblastic leukaemia: long-term follow-up of a prospective, randomised, multicentre trial. *The Lancet Oncology*.

[141] Tebbi C. K., London W. B., Friedman D. (2007). Dexrazoxane-associated risk for acute myeloid leukemia/myelodysplastic syndrome and other secondary malignancies in pediatric Hodgkin’s disease. *Journal of Clinical Oncology*.

[142] http://www.ema.europa.eu/docs/en_GB/document_library/Referrals_document/Dexrazoxane_31/WC500108011.pdf

[143] Hensley M. L., Hagerty K. L., Kewalramani T. (2009). American Society of Clinical Oncology 2008 clinical practice guideline update: use of chemotherapy and radiation therapy protectants. *Journal of Clinical Oncology*.

[144] Simůnek T., Stérba M., Popelová O., Adamcová M., Hrdina R., Gersl V. (2009). Anthracycline-induced cardiotoxicity: overview of studies examining the roles of oxidative stress and free cellular iron. *Pharmacological Reports*.

[145] Lyu Y. L., Kerrigan J. E., Lin C. P. (2007). Topoisomerase IIβ mediated DNA double-strand breaks: implications in doxorubicin cardiotoxicity and prevention by dexrazoxane. *Cancer Research*.

[146] Jirkovská-Vávrová A., Roh J., Lenčová-Popelová O. (2015). Synthesis and analysis of novel analogues of dexrazoxane and its open-ring hydrolysis product for protection against anthracycline cardiotoxicity *in vitro* and *in vivo*. *Toxicology Research*.

[147] Kalay N., Basar E., Ozdogru I. (2006). Protective effects of carvedilol against anthracycline-induced cardiomyopathy. *Journal of the American College of Cardiology*.

[148] Kaya M. G., Ozkan M., Gunebakmaz O. (2013). Protective effects of nebivolol against anthracycline-induced cardiomyopathy: a randomized control study. *International Journal of Cardiology*.

[149] El-Shitany N. A., Tolba O. A., El-Shanshory M. R., El-Hawary E. E. (2012). Protective effect of carvedilol on adriamycin-induced left ventricular dysfunction in children with acute lymphoblastic leukemia. *Journal of Cardiac Failure*.

[150] Mason R. P., Kalinowski L., Jacob R. F., Jacoby A. M., Malinski T. (2005). Nebivolol reduces nitroxidative stress and restores nitric oxide bioavailability in endothelium of Black Americans. *Circulation*.

[151] Bosch X., Rovira M., Sitges M. (2013). Enalapril and carvedilol for preventing chemotherapy-induced left ventricular systolic dysfunction in patients with malignant hemopathies: the OVERCOME trial. *Journal of the American College of Cardiology*.

[152] Cardinale D., Colombo A., Lamantia G. (2010). Anthracycline-induced cardiomyopathy: clinical relevance and response to pharmacologic therapy. *Journal of the American College of Cardiology*.

[153] Zhou Q., Liao J. K. (2010). Pleiotropic effects of statins: basic research and clinical perspectives. *Circulation Journal*.

[154] Riad A., Bien S., Westermann D. (2009). Pretreatment with statin attenuates the cardiotoxicity of doxorubicin in mice. *Cancer Research*.

[155] Henninger C., Huelsenbeck S., Wenzel P. (2015). Chronic heart damage following doxorubicin treatment is alleviated by lovastatin. *Pharmacological Research*.

[156] Acar Z., Kale A., Turgut M. (2011). Efficiency of atorvastatin in the protection of anthracycline-induced cardiomyopathy. *Journal of the American College of Cardiology*.

[157] Seicean S., Seicean A., Plana J. C., Budd G. T., Marwick T. H. (2012). Effect of statin therapy on the risk for incident heart failure in patients with breast cancer receiving anthracycline chemotherapy: an observational clinical cohort study. *Journal of the American College of Cardiology*.

[158] Chotenimitkhun R., D’Agostino R., Lawrence J. A. (2015). Chronic statin administration may attenuate early anthracycline-associated declines in left ventricular ejection function. *The Canadian Journal of Cardiology*.

[159] Egea J., Fabregat I., Frapart Y. M. (2017). European contribution to the study of ROS: A summary of the findings and prospects for the future from the COST action BM1203 (EU-ROS). *Redox Biology*.

[160] Brown D. I., Griendling K. K. (2015). Regulation of signal transduction by reactive oxygen species in the cardiovascular system. *Circulation Research*.

[161] Cai H. (2005). Hydrogen peroxide regulation of endothelial function: origins, mechanisms, and consequences. *Cardiovascular Research*.

[162] Song M., Chen Y., Gong G., Murphy E., Rabinovitch P. S., Dorn G. W. (2014). Super-suppression of mitochondrial reactive oxygen species signaling impairs compensatory autophagy in primary mitophagic cardiomyopathy. *Circulation Research*.

[163] Görlach A., Dimova E. Y., Petry A. (2015). Reactive oxygen species, nutrition, hypoxia and diseases: problems solved?. *Redox Biology*.

[164] Bigarella C. L., Liang R., Ghaffari S. (2014). Stem cells and the impact of ROS signaling. *Development*.

[165] Gomez-Cabrera M. C., Ristow M., Viña J. (2012). Antioxidant supplements in exercise: worse than useless?. *American Journal of Physiology, Endocrinology and Metabolism*.

